# Reimagining Tuberculosis Control in the Era of Genomics: The Case for Global Investment in *Mycobacterium tuberculosis* Genomic Surveillance

**DOI:** 10.3390/pathogens14100975

**Published:** 2025-09-26

**Authors:** Gerald Mboowa

**Affiliations:** 1 Broad Institute of MIT and Harvard, 415 Main Street, Cambridge, MA 02142, USA; gmboowa@gmail.com; 2 African Centre of Excellence in Bioinformatics and Data-Intensive Sciences, Infectious Diseases Institute, College of Health Sciences, Makerere University, Kampala P.O. Box 22418, Uganda

**Keywords:** tuberculosis, genomic surveillance, machine learning, drug resistance, whole-genome sequencing, low-and middle-income countries (LMICs), public health genomics

## Abstract

Drug-resistant *Mycobacterium tuberculosis* remains a significant global public health threat. While whole-genome sequencing (WGS) holds immense promise for understanding transmission dynamics and drug resistance mechanisms, its integration into routine surveillance remains limited. Additionally, insights from WGS are increasingly contributing to vaccine discovery by identifying novel antigenic targets and understanding pathogen evolution. The COVID-19 pandemic catalyzed an unprecedented expansion of genomic capacity in many low- and middle-income countries (LMICs), with public health institutions acquiring next-generation sequencing (NGS) platforms and developing local expertise in real-time pathogen surveillance. This hard-won capacity now represents a transformative opportunity to accelerate TB control enabling rapid detection of drug-resistant strains and high-resolution mapping of transmission networks that are critical for timely, targeted interventions. Furthermore, the integration of machine learning with genomic and clinical data offers a powerful avenue to improve the prediction of drug resistance and to tailor patient-specific TB management strategies. This article examines the practical challenges, emerging opportunities, and policy considerations necessary to embed genomic epidemiology within national TB control programs, particularly in high-burden, resource-constrained settings.

## 1. Introduction

Multidrug-resistant and rifampicin-resistant tuberculosis (MDR/RR-TB) represents a persistent and severe obstacle to global TB control, with profound implications for treatment outcomes and public health. Although recent years have seen modest declines, the global burden remains substantial, with approximately 3.3% of new TB cases and 17% of previously treated cases exhibiting resistance to rifampicin or multiple first-line drugs [1]. These figures highlight the persistent challenge of drug resistance in TB care and underscore the urgent need for improved diagnostics, treatment, and surveillance strategies to effectively address MDR/RR-TB. Treatment for drug-resistant tuberculosis (DR-TB), which involves the use of second-line anti-TB drugs, is often associated with severe adverse effects, some of which can be life-threatening [2]. MDR and extensively drug-resistant (XDR) TB not only compromise treatment efficacy and prolong treatment duration, but also reduce patients’ quality of life, increase mortality rates, strain healthcare systems, elevate treatment costs, and facilitate the continued transmission of resistant strains within communities. In high-burden settings, a person with untreated active TB may transmit infection to 10–15 individuals over the course of a year [3]. In recent years, genomic tools have shown great promise as valuable allies in the fight against TB, with growing applications in rapid and accurate detection of TB, identification of drug resistance mutations, and providing critical insights into transmission dynamics, outbreak origins, and evolutionary patterns. However, their uptake remains uneven globally, with most applications concentrated in high-resource or research-intensive settings. When combined with emerging data science approaches such as machine learning (ML), genomic tools not only accelerate data processing and analysis but also substantially strengthen the interpretive and predictive value of genomic information. This integration enables early detection of drug resistance, fine-scale mapping of transmission networks, real-time outbreak monitoring, and risk stratification for targeted interventions. It further supports the development of predictive models for disease progression and treatment response, thereby advancing precision public health strategies, an especially critical need in high-burden, resource-limited TB settings where timely, data-driven decisions can yield the greatest impact.

**Figure 1:**
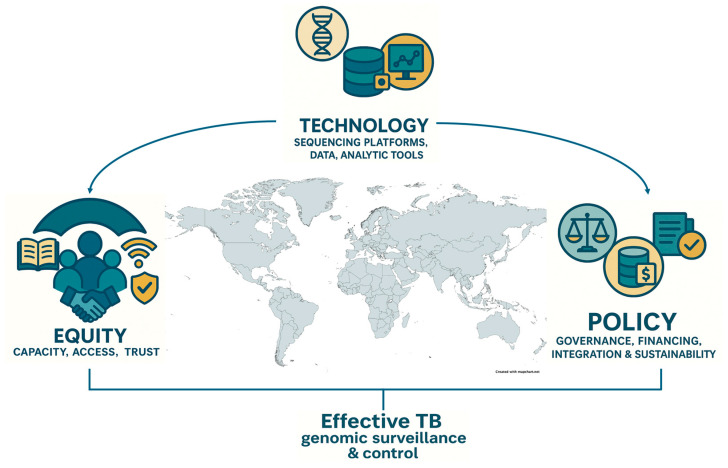
Conceptual framework linking three foundational pillars—technology, policy, and equity—that underpin effective TB genomic surveillance and control. Technology encompasses sequencing platforms, analytical tools, and data systems; policy refers to governance frameworks, sustainable financing, and integration into national TB programs; and equity emphasizes capacity building, access, and trust to ensure inclusive benefits across regions. The convergence of these pillars enables robust genomic surveillance and informs public health action against tuberculosis in diverse global contexts.

Despite substantial technological progress, genomic surveillance of Mycobacterium tuberculosis remains underutilized in high-burden countries and is seldom embedded within routine public health programs. Widespread implementation is hindered by persistent barriers, including the high cost of sequencing, limited laboratory infrastructure, shortages of skilled bioinformatics personnel, inadequate analytical capacity, and fragmented or absent policy frameworks. Recent economic evaluations provide encouraging evidence that routine sequencing can be both feasible and cost-effective in low- and middle-income country (LMIC) settings. For example, a multi-country study of targeted next-generation sequencing (tNGS) for TB drug resistance in India, South Africa, and Georgia reported that tNGS was cost-saving and health-improving in India, cost-effective in South Africa (incremental cost-effectiveness ratio [ICER]: USD 15,619 per disability-adjusted life year [DALY] averted at a willingness-to-pay threshold of USD 21,165), though less favorable under current conditions in Georgia [4]. Importantly, scenario analyses showed that reducing per-test costs by 50% rendered tNGS cost-effective across all three countries, underscoring how scale-up, batching efficiencies, and multi-pathogen sequencing applications can substantially improve value for money. Moreover, these findings highlight the potential for a strong return on investment, as upfront investments in sequencing capacity can yield downstream savings through more efficient diagnosis, optimized treatment allocation, and reduced transmission. Additionally, they highlight related modeling and budget impact studies which emphasize that per-test cost, batching efficiency, and health system infrastructure strongly influence cost-effectiveness outcomes [4]. Taken together, these analyses indicate that while routine Whole-genome sequencing (WGS)/tNGS implementation requires upfront investment, it can be cost-effective and health-improving in LMIC contexts, particularly when linked to improved turnaround time, reduced loss to follow-up, and multi-pathogen sequencing applications. Additionally, concerns around data governance, sustainability, and integration with existing surveillance systems further limit its adoption. However, the COVID-19 pandemic demonstrated that rapid scaling of genomic technologies is achievable [5] in LMICs when backed by strong political will, coordinated partnerships, and sustained financial investment (Figure 1). These hard-earned lessons must now be translated into action for TB control in these settings, where genomics can play a transformative role in detecting drug resistance, tracking transmission, and guiding precision public health interventions.

## 2. Global Distribution of MTBC Lineages and Implications for Genomic Surveillance

**Figure 2:**
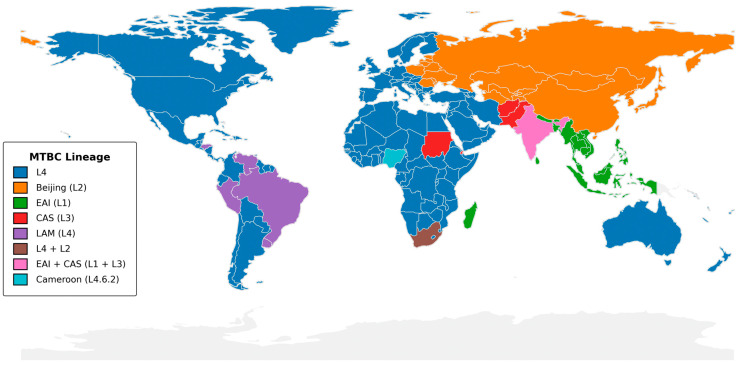
Global distribution of dominant *Mycobacterium tuberculosis* complex lineages. The map indicates the most prevalent MTBC lineage in each country, using SNP-based nomenclature as defined by Coll et al. [18]. Lineage assignments were drawn from WHO Global Tuberculosis Reports (2020–2023) [19], the CRyPTIC Consortium study [20], and national TB surveillance reports. Country boundaries are from Natural Earth v1:110 m. The figure was generated in Python 3.11 (GeoPandas v0.12.2, Matplotlib v3.7.1) with shapefile processing in QGIS v3.28; colors were selected to be accessible under color-vision deficiency. Only the dominant lineage(s) per country is/are shown.

A comprehensive view of dominant *Mycobacterium tuberculosis* complex (MTBC) lineages reveals stark geographic patterns that have direct implications for national TB control programs (Figure 2). Across North America, Europe, and much of Sub-Saharan Africa, the Euro-American Lineage 4 (L4) predominates [6], reflecting both historical dissemination via colonial trade routes and ongoing transmission within high-burden settings [6]. Within Latin America, the LAM sub-clade of L4 remains dominant in Brazil, Peru, Uruguay, and extending through Central America and the Caribbean, whereas the specialized “Cameroon” sub-lineage (L4.6.2) accounts for majority of cases in Nigeria [7].

In contrast, East Asia and parts of Eastern Europe are marked by a high prevalence of the Beijing lineage (L2), which is associated with rapid transmission and elevated rates of multidrug resistance [8]. South Asia displays a mosaic of Lineage 1 (EAI) and Lineage 3 (CAS), particularly in India and neighboring countries, underscoring the value of dual-targeted sequencing approaches. Smaller pockets such as the Sudan and the Arabian Peninsula feature the CAS lineage (L3), while islands like Madagascar and parts of Southeast Asia retain strong EAI (L1) signals.

These lineage distributions highlight three immediate priorities for TB control programs:

### 2.1. DR-TB Sequencing Panel Design

Regions dominated by L2 (Beijing) can incorporate probes for rapid detection of known resistance mutations associated with this lineage, whereas L4-focused panels remain updated to capture emerging LAM, Haarlem, and T-family markers [9].

### 2.2. Decentralized Genomic Capacity

High-burden L4 countries where genomic diversity spans multiple sub-clades need investment in both short-read and long-read platforms to resolve complex transmission networks. In contrast, areas with more homogeneous lineage profiles may benefit from tNGS assays that can be deployed in peripheral laboratories.

### 2.3. Geo-Stratified Data Integration and Policy Development

National TB control programs should link lineage-specific resistance profiles to tailored treatment algorithms and contact-tracing protocols. For example, the known association of Beijing strains with higher rates of rifampicin resistance warrants enhanced surveillance and rapid molecular diagnostics in East Asia [10,11,12,13,14,15,16]. Likewise, the predominance of LAM in Latin America [17] suggests utility in pan-regional data sharing initiatives to track cross-border transmission.

By aligning genomic surveillance strategies with these geospatial lineage insights, TB control programs can optimize resource allocation, accelerate outbreak detection, and refine individualized therapy thereby translating pathogen genomics into actionable public health impact.

## 3. Value of Genomics in TB Control

WGS is a transformative tool for TB control, providing comprehensive insights into the biology, evolution, transmission dynamics, and drug resistance mechanisms of *Mycobacterium tuberculosis*. Its greatest return on investment is realized in applications where high-resolution data are indispensable such as outbreak investigations, surveillance of MDR TB, and distinguishing relapse from reinfection, whereas other diagnostic tools, such as Xpert^®^ MTB/RIF, remain more cost-effective for routine case detection in many settings. One of the most impactful applications of WGS is the rapid and accurate detection of genomic mutations associated with resistance to key anti-TB drugs, including rifampicin, isoniazid, fluoroquinolones, and second-line injectable agents. This capability enables early, individualized treatment optimization, thereby minimizing delays, reducing transmission, and improving clinical outcomes. Beyond drug resistance profiling, WGS supports high-resolution phylogenetic analysis to delineate transmission networks, identify outbreak sources, and differentiate relapse from reinfection. It also contributes to the discovery of novel diagnostic markers, informs vaccine and therapeutic target development, and enhances surveillance by detecting emerging resistance patterns in near real time. Integrating WGS into routine TB control programs can substantially accelerate the global response to DR TB, particularly when paired with robust data systems, bioinformatics capacity, and actionable clinical reporting frameworks. Practical software options for routine TB genomics emphasizing offline use, open-source status, and maintenance are summarized in Table 1. Such detail is essential for guiding public health interventions and contact tracing strategies. Furthermore, genomic data enable precise strain typing and lineage classification, uncovering lineage-specific trends in drug resistance, virulence, and pathogenicity. For instance, Lineage 2 (Beijing) strains are consistently associated with higher transmissibility, increased virulence, and a greater propensity for developing drug resistance [2]. These insights are essential for understanding the evolution and spread of TB in different populations and geographic settings.

WGS of diverse *Mycobacterium tuberculosis* isolates collected over multiple years and across geographically and epidemiologically distinct regions holds immense promise for accelerating TB vaccine development. Analysis of this global genomic diversity enables the identification of conserved antigens, immune evasion mechanisms, lineage-specific adaptations, and genetic signatures of virulence that are critical for designing novel, broadly protective vaccines. Such genomic approaches support the prioritization of vaccine targets that are functionally essential, consistently expressed, and less prone to mutation characteristics that help preserve efficacy across different *Mycobacterium tuberculosis* lineages, diverse transmission settings, high HIV-prevalence populations, and individuals with latent TB infection. Comparative genomics further provides insights into how *Mycobacterium tuberculosis* adapts under immune and therapeutic pressures, informing the selection of durable antigens. 

Importantly, the global repository of *Mycobacterium tuberculosis* genomic data has expanded substantially, with over 290,000 entries now available in the NCBI Sequence Read Archive (SRA) [31]. This makes *Mycobacterium tuberculosis* the most sequenced bacterial pathogen worldwide and the third most sequenced pathogen overall after SARS-CoV-2 and influenza viruses.

Harnessing this wealth of data through integrative genomic, immunological, reverse vaccinology [32], and bioinformatic approaches could be pivotal in informing next-generation TB vaccines that are effective across diverse populations, age groups, and disease stages. The sequencing of over 17.4 million SARS-CoV-2 virus genomes [33] facilitated the rapid development of highly effective vaccines using innovative technologies such as mRNA platforms. This achievement was made possible through coordinated global efforts to collect and sequence samples across diverse geographic regions and over time, ensuring robust representation of circulating viral variants and enabling real-time tracking of viral evolution and transmission dynamics. Drawing inspiration from this success, a similarly concerted global effort in TB genomics and sequencing could accelerate tuberculosis vaccine development. Given that both pathogens are transmitted via aerosols and primarily infect the respiratory tract, leveraging the extensive genomic data available for *Mycobacterium tuberculosis* offers significant potential for rational vaccine design [32]. Such approaches can enable the development of next-generation TB vaccines that are tailored to the pathogen’s genetic diversity and evolutionary dynamics.

An important catalyst of the COVID-19 pandemic has been the rapid deployment and expansion of NGS platforms across numerous public health laboratories worldwide, including in many high TB burden settings. These newly established sequencing capacities, initially scaled to support SARS-CoV-2 surveillance, now provide a robust foundation that can be leveraged to strengthen disease control programs for other major infectious diseases, including Malaria, HIV, and Tuberculosis [5]. This expanded genomic infrastructure enhances the ability to detect pathogens and their antimicrobial resistance profiles with greater speed and precision, supporting more timely and targeted public health responses. Equally important, however, is sustained investment in maintaining and integrating this genomic capacity into routine surveillance systems. Doing so is critical not only for ongoing disease control but also for the early detection of emerging and re-emerging pathogens, particularly in transmission hotspots and vulnerable populations where rapid response can significantly mitigate epidemic spread and improve health outcomes. Moreover, sustaining this multi-pathogen capacity and expertise will equip public health institutions with the capability to detect even novel, previously unknown pathogens (‘*pathogen X*’) with epidemic or pandemic potential in real time, thereby strengthening global preparedness for future health threats.

Tuberculosis is caused by members of the MTBC, a group of several closely related species such as includes *M. tuberculosis*, *M. africanum*, *M. bovis*, *M. caprae*, *M. microti*, *M. pinnipedii*, *M. canettii*, *M. mung*, *M. orygis*, and *M. suricattae* [34,35]. These species exhibit differences in virulence, geographic distribution, host range, and epidemiological significance, with some predominantly infecting humans (e.g., *M. tuberculosis*, *M. africanum*), while others primarily affect animals with occasional zoonotic transmission (e.g., *M. bovis*, *M. orygis*, *M. pinnipedii*) [36]. WGS provides the high resolution necessary to accurately distinguish between species within the MTBC and, importantly, to detect mixed infections involving multiple MTBC strains within a single host. Epidemiological studies estimate that approximately 10–20% of individuals in high TB burden settings harbor infections with more than one MTB strain simultaneously [37,38]. Mixed infections often go undetected by conventional diagnostic methods yet may have important clinical consequences, as co-infecting strains can differ in drug resistance profiles and treatment responses. For instance, *Mycobacterium bovis*, a member of the MTBC, is intrinsically resistant to pyrazinamide, a cornerstone first-line anti-TB drug, underscoring the importance of precise species-level identification to guide effective therapy. By enabling the detection of such complex infections, WGS supports the design of more tailored treatment regimens, reduces the risk of treatment failure, and ultimately improves clinical outcomes for TB patients.

## 4. Integrating Machine Learning Across Clinical, Imaging, and Genomic Data in TB Control

Building on the strengths of WGS in TB control, emerging data science approaches such as ML are increasingly being integrated to enhance the predictive power of genomic data. While WGS provides comprehensive insights into the genomic architecture of *Mycobacterium tuberculosis*, ML models can detect subtle and complex patterns often beyond the reach of rule-based approaches and link them to host characteristics, epidemiological trends, and clinical outcomes. ML also enables the discovery of genomic and transcriptomic signatures associated with host–pathogen interactions, including transcriptional responses of *Mycobacterium tuberculosis* to drug pressure, immune status, and co-infections such as HIV, COVID-19, and chronic obstructive pulmonary disease (COPD). By integrating genomic and transcriptomic datasets, ML facilitates a systems-level understanding of pathogen adaptation across diverse biological and epidemiological contexts. These approaches are particularly powerful in light of the rapidly expanding *Mycobacterium tuberculosis* sequencing and expression datasets from global cohorts, enabling models to improve the accuracy of resistance prediction, uncover dynamic regulatory mechanisms, and support context-specific treatment strategies. Ultimately, this integrative framework strengthens precision in both public health decision-making and clinical interventions across different phases of the TB epidemic.

In a Ugandan cohort of 226 *Mycobacterium tuberculosis* isolates, 182 passed quality control and were analyzed. Models were trained and selected with five-fold cross-validation, then evaluated for generalizability on an independent South African set (*n* = 236). For each drug, sensitivity, specificity, Matthews correlation coefficient (MCC), and area under the curve (AUC) were estimated with 95% confidence intervals [39]. Logistic regression achieved high sensitivity for rifampicin resistance, whereas gradient boosting and XGBoost showed high specificity for isoniazid and ethambutol, respectively [39]. HIV status emerged as a significant clinical covariate, supporting the integration of host metadata into genomic models particularly relevant in high TB/HIV-burden settings. A head-to-head comparison revealed complementary strengths: TB-Profiler outperformed ML models across most drugs in the external South African set, reflecting the portability of curated mutation catalogs, while locally trained ML models performed comparably and for several drugs (e.g., isoniazid, fluoroquinolones) better on the Ugandan data, capturing population-specific genomic and epidemiological signatures not fully represented in rule-based resources. For drugs with well-characterized resistance determinants, TB-Profiler retained an advantage in external validation; where mechanisms are heterogeneous or incompletely cataloged, ML provided additional predictive power. Together, these findings underscore the value of contextual, locally trained models alongside continued use of curated rule-based tools for broader generalizability [28,29]. Current bioinformatics tools for predicting genotypic drug resistance in *Mycobacterium tuberculosis* primarily rely on curated reference databases that map known resistance-associated mutations to phenotypic profiles for first- and second-line drugs. In contrast, emerging ML approaches can move beyond predefined mutation catalogs, identifying novel markers and complex patterns across diverse data types to predict a wider range of TB outcomes [21]. Recent studies have demonstrated the utility of ML for identifying plasma proteomic biosignatures that can improve TB screening in children [40]. By analyzing high-throughput proteomic data across diverse settings, researchers have developed predictive models with AUCs of 0.87–0.88 that meet WHO target product profiles for TB screening tests [41], underscoring the potential of ML to support non-sputum-based TB diagnostics in pediatric population [40].

**Figure 3:**
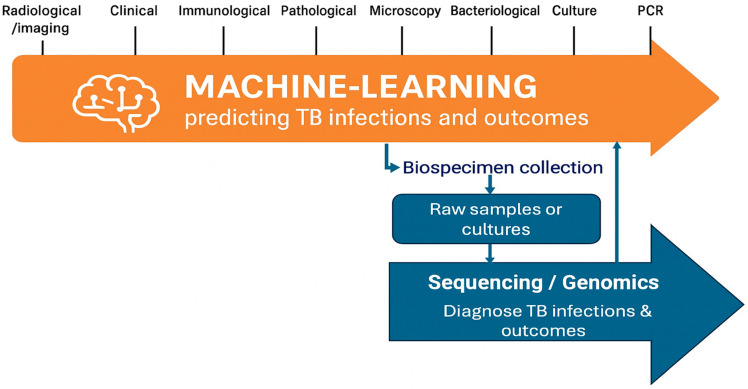
Integration of ML and genomics in TB management. The figure illustrates the continuum of diagnostic and predictive data types relevant to TB control, encompassing radiological, clinical, immunological, microbiological, and molecular diagnostic information. ML methods leverage this diverse data spectrum to predict infection status and clinical outcomes, while genomics and bioinformatics approaches operate downstream beginning with biospecimen collection and sequencing to enable TB diagnosis and drug resistance characterization.

Effective TB management relies on the integration of diverse data types that can support screening, prediction of suspected cases and definitive diagnosis. These data sources span radiological and imaging findings (e.g., chest X-rays, CT scans), clinical symptoms and history, hematological and biochemical markers, immunological responses, pathological assessments, and microbiological methods including microscopy, culture, and drug susceptibility testing. Molecular diagnostic approaches such as polymerase chain reaction (PCR) and WGS further enhance diagnostic precision by enabling the detection of *Mycobacterium tuberculosis* and profiling of drug resistance. Integrating these heterogeneous data streams offers a comprehensive view of TB infection, improves diagnostic accuracy, and supports tailored treatment strategies. ML approaches are uniquely positioned to harness this diverse data landscape across the full spectrum of TB continuum (Figure 3). While genomics and bioinformatics workflows typically begin with a collected biospecimen and play a critical role in confirming TB diagnosis and elucidating drug resistance mechanisms, their application is often narrowly focused on pathogen-specific genomic information. In contrast, ML approaches can integrate heterogeneous data types including radiological, clinical, hematological, immunological, microbiological, and genomic data across various diagnostic modalities and disease stages. Recent evaluations demonstrate that ML models trained on chest X-ray datasets (e.g., CAD4TB, qXR, INSIGHT CXR) achieve sensitivities around 90%, with CAD4TB achieving ~ 73.8% specificity at 90% sensitivity (AUC 0.774–0.819) in a multi-country head-to-head study [42]. In real-world settings such as Gaza, CAD4TBv6 yielded 80.2% sensitivity and 94.0% specificity at threshold > 40 [43]. Systematic review evidence further indicates that deep-learning CAD systems generally outperform traditional ML (AUC ~ 0.91 vs. 0.82), although clinical validation lag behind development-phase performance (AUC 0.75 in clinical studies) [44]. In genomic applications, ML models including logistic regression, XGBoost, and CNNs have demonstrated high accuracy in predicting resistance directly from WGS (e.g., rifampicin AUC up to 0.96) [45,46]. A global multi-drug classifier achieved F1 scores of 81–94%, outperforming rule-based tools like Mykrobe [22]; large-scale WGS ML pipelines similarly showed robust prediction of resistance phenotypes and MICs [20]. However, these models’ generalizability can be limited. Deep-learning CXR models show reduced external performance when trained and applied across distinct populations [43,45], and systematic reviews highlight the paucity of clinical-validation studies [44]. Broader ML literature warns of risks related to bias, fairness, and overfitting without rigorous external validation [47]. This enables more holistic prediction of TB infection risk, disease progression, and treatment outcomes, supporting earlier interventions and more personalized patient care. These findings suggest that, while ML may not replace established rule-based tools such as TB-Profiler [21], it serves as a powerful complementary approach. By integrating both genomic and clinical data, ML offers promising opportunities to strengthen TB surveillance, guide individualized therapy, and improve outcomes, particularly in high-burden, resource-constrained settings.

To synthesize the current evidence base, Table 2 highlights representative top-performing ML models for TB drug-resistance prediction, showing the input features, drugs predicted, performance metrics, and the key predictors identified.

## 5. Barriers to Widespread Adoption

In 2024, the WHO issued updated consolidated guidelines on TB diagnosis, which included recommendations supporting the use of tNGS for detecting drug-resistant TB [52], marking a pivotal advancement in global TB diagnostics. However, realizing the full potential of genomic technologies in TB control requires a deliberate and equitable approach to implementation. Despite the promise of WGS and genomic epidemiology to revolutionize diagnosis, treatment, and surveillance, several systemic and operational barriers continue to hinder their widespread adoption in TB high-burden LMICs. To bridge this gap, genomics must be deployed through a lens of equity guided by foundational pillars that include decentralized capacity building, access to open-source tools and data platforms, and integration into national policy and programmatic frameworks.

Case studies from India [53], South Africa [54,55], and Uganda [56] highlight both common and context-specific challenges. In India, pilot programs integrating WGS into national TB control demonstrated feasibility but also underscored persistent barriers related to reagent costs, centralized infrastructure, and limited capacity for large-scale data analysis. In South Africa, where WGS has been implemented in a subset of reference laboratories, challenges include high operational costs, sustainability of funding, and difficulties in maintaining uninterrupted supply chains for sequencing consumables. The Ugandan experience illustrates the dual challenge of financial constraints and limited bioinformatics expertise, which restrict the ability of laboratories to move beyond research projects toward routine public health integration.

Across these contexts, additional barriers include fragmented data-sharing frameworks, inadequate internet connectivity for timely database updates, and limited local availability of trained personnel to manage both sequencing platforms and downstream bioinformatics pipelines. These challenges are further compounded by the relatively high cost of equipment servicing and the dependency on international suppliers for critical consumables. Collectively, these case studies demonstrate that while WGS has clear potential for strengthening TB programs, successful implementation in LMICs requires more than capital investment in sequencing platforms. Coordinated efforts to build regional sequencing hubs, invest in training and workforce development, and establish supportive policy frameworks will be critical to overcoming these barriers and ensuring equitable access to genomic technologies. 

Governance models for national program integration: Embedding WGS into national TB control programs requires clear and accountable governance models to ensure sustainability and impact. Effective models typically establish a multisectoral coordination mechanism that includes ministries of health, national TB control programs, national public health institutes, research/academic partners, and implementing laboratories. These structures define roles and responsibilities across the sequencing pipeline from sample referral, data generation, data access/sharing and bioinformatics analysis to reporting and translation into programmatic decision-making. Governance frameworks also specify standards for data sharing, protection of patient confidentiality, and integration with national health information systems. In addition, successful governance models embed WGS within existing national TB strategic plans, supported by line-item budgeting, domestic financing commitments, and standardized operating procedures endorsed by ministries of health. This alignment ensures that WGS is not treated as a stand-alone research activity but as a core surveillance and diagnostic function with clear accountability and measurable impact.

Infrastructure, supply chain and cost: The relatively high cost of next-generation sequencing platforms [57] and reagents remains a significant barrier to widespread adoption in many LMICs. In most countries, sequencing infrastructure is concentrated in national reference laboratories or a few well-resourced academic institutions, limiting access for decentralized public health programs. This centralization contributes to logistical bottlenecks, including delays in sample transport, prolonged turnaround times, and fragmented data workflows, all of which compromise the timeliness and public health utility of genomic data for TB surveillance and response.

Beyond the initial capital investment, sustaining sequencing operations requires continuous expenditures for equipment maintenance, reagent procurement, software licenses, and platform calibration costs that are often prohibitive in settings with constrained domestic health budgets. These financial challenges are compounded by frequent supply chain disruptions, such as customs delays, dependency on international suppliers, and limited local availability of specialized consumables. From a computational perspective, downstream data analysis requires access to robust processing units (CPUs and, in some cases, GPUs for ML applications), reliable internet connectivity for software/database updates, and adequate secure data storage infrastructure. Many LMIC laboratories face bandwidth limitations and restricted on-site storage capacity, further hindering the integration of genomic technologies into routine workflows. Collectively, these financial, infrastructural, and computational constraints hinder the routine adoption of sequencing for national TB control programs and widen the gap in genomic equity between high-income and resource-limited settings. Addressing these barriers will require coordinated investments in both laboratory and digital infrastructure, regional capacity sharing (e.g., cloud-based or hub-and-spoke models), and supportive policy frameworks that ensure sustainable implementation and equitable access to pathogen genomics.

Data sharing challenges: Effective genomic surveillance depends on timely, transparent, and interoperable data sharing among laboratories, institutions, and countries. However, persistent challenges undermine this ideal, including concerns about data sovereignty, unclear governance frameworks, and apprehensions around intellectual property rights and patient confidentiality. Many countries, particularly in low-resource settings, lack national policies, legal instruments, or digital infrastructure to facilitate secure, standardized, and ethically sound data exchange. The absence of clear data stewardship protocols not only hinders cross-border genomic collaboration but also reduces the potential for real-time threat detection, comparative analyses, and globally coordinated responses. These gaps are especially problematic in the context of transnational health threats like DR TB, where delays in data sharing can impede containment efforts. Protective approaches include adopting equitable data governance frameworks that balance national sovereignty with global health security, such as those promoted by WHO and Public Health Alliance for Genomic Epidemiology (PHA4GE); establishing clear data use agreements and material transfer policies to protect patient confidentiality and intellectual property; and developing secure, federated data-sharing infrastructures that allow countries to retain control of sensitive data while enabling international collaboration [58,59]. Best practices also emphasize building trust through transparent benefit-sharing mechanisms, ensuring that local researchers are decision-makers in collaborative projects, and aligning data-sharing initiatives with national legal frameworks [60,61]. Addressing these challenges requires the development of equitable and inclusive governance frameworks that balance open science with national interests, ensure trust, and promote responsible data sharing across regions and sectors.

DR TB analysis: While sequencing technologies have become increasingly accessible [57], data analysis remains a major bottleneck in the implementation of genomic surveillance for tuberculosis. National TB control programs in many LMICs face a widespread shortage of trained bioinformaticians and data scientists, leading to heavy reliance on external collaborators or, in many cases, underutilization of the *Mycobacterium tuberculosis* genomic data generated. Interpreting complex sequencing outputs requires a multidisciplinary skill set that spans microbial genomics, bioinformatics, epidemiology, and data science expertise that is often scarce or fragmented in resource-limited settings. This capacity gap undermines the potential of WGS as a real-time, decision-support tool for TB control. Mitigation strategies include embedding bioinformatics training into national TB control program capacity-building efforts; leveraging regional training hubs for ongoing workforce development; and adopting open-source, user-friendly tools such as Mykrobe and TB-Profiler, which reduce reliance on highly specialized expertise [62]. Sustaining staff capacity requires deliberate measures such as creating defined career pathways within national TB control programs, providing competitive retention incentives, and institutionalizing continuous professional development opportunities. Embedding bioinformatics roles into government staffing structures, rather than short-term project-based contracts, will further ensure long-term workforce stability and prevent attrition of trained personnel. In addition, retention strategies such as regional training networks, continuous professional development, diversified funding streams, and incentives to mitigate brain drain are critical to maintaining a resilient workforce 

There is growing consensus around the use of validated, open-source TB WGS analysis tools such as Mykrobe and TB-Profiler, which provide standardized, user-friendly outputs for drug resistance prediction and lineage classification. These tools can be deployed locally, enabling countries to retain data sovereignty and perform analyses offline, an important advantage in settings with limited or unreliable internet connectivity. However, open-source solutions alone do not resolve equity barriers. Their benefits can only be realized when paired with adequate computational infrastructure, reliable internet connectivity, skilled personnel, and supportive policy frameworks that ensure sustained integration into national TB control programs. Moreover, to sustain their diagnostic utility, it is critical that the underlying reference databases are regularly updated to reflect newly identified resistance-conferring mutations, novel lineages, and emerging trends, especially as new TB drugs are rolled out. Without timely updates, the accuracy of resistance predictions may decline, compromising the early detection of resistance and the ability to respond to evolving drug resistance landscapes. Mitigation strategies here include institutionalizing regular database updates via global consortia such as ReSeqTB, ensuring interoperability with the most updated WHO’s TB Mutation Catalogue, and encouraging LMIC laboratories to participate in shared curation exercises. Best practices also recommend developing sustainable funding mechanisms to maintain bioinformatics infrastructure and creating multi-stakeholder partnerships that include governments, academia, and donors to ensure long-term viability. Building long-term, in-country capacity for bioinformatics training, infrastructure, and database curation is therefore essential for ensuring that *Mycobacterium tuberculosis* genomic data can be effectively interpreted and translated into public health action.

Financing models and sustainability: Sustainable integration of WGS into TB programs requires not only technological readiness but also robust financing frameworks. In most LMICs, the high initial capital costs for sequencing platforms, combined with recurrent expenditures for reagents, maintenance, and bioinformatics support, necessitate diversified funding strategies. Public–private partnerships (PPPs) have shown promise in bridging these gaps for example, through cost-sharing agreements with diagnostic companies, philanthropic support for reagent subsidies, or collaborations with cloud service providers to reduce computational infrastructure costs. National budgeting strategies must also evolve to integrate sequencing within broader TB control program funding streams, ensuring that genomic surveillance is not treated as a stand-alone research activity but as a core public health function. Innovative approaches such as pooled procurement of reagents and consumables at regional or continental levels could further reduce costs through economies of scale. Ultimately, long-term sustainability will depend on a blended model combining domestic financing, catalytic donor support, and PPP-driven innovation, embedded within national TB control budgets. Without this deliberate financial planning, WGS risks remaining a pilot-level innovation rather than a routine tool in high-burden settings.

Regulatory uncertainty: The absence of standardized protocols for sequencing, data analysis, and result interpretation across countries continues to pose a significant regulatory and operational challenge to the broader integration of WGS into TB control efforts. In many settings, national TB programs have not yet formally incorporated WGS into diagnostic or surveillance workflows. This lag is partly attributed to limited technical support following the WHO’s endorsement of tNGS for drug resistance detection, as well as the lack of clear operational frameworks, implementation guidance, and large-scale validation studies that demonstrate effectiveness in real-world settings.

Furthermore, there is a critical need to harmonize the use of sequencing panels by distinguishing those developed for research purposes from those validated for clinical diagnostics. This includes the establishment of standardized procedures for panel validation, clear criteria for performance assessment, and a well-defined role for regulatory authorities whether national regulatory agencies, the WHO, or other global entities in approving and endorsing sequencing-based diagnostic platforms. The lack of globally recognized standards and policies hampers the clinical translation of genomic data, often relegating it to academic or research contexts rather than integrating it into national TB control programs where it could inform real-time decision-making and public health response.

To unlock the full potential of WGS in TB control, coordinated efforts are needed to establish regulatory pathways, promote quality assurance and proficiency testing, and align global and national policies on genomic data use. Implementation science can play a vital role in generating the evidence needed to support policy decisions and accelerate the adoption of WGS into routine public health practice.

Dynamic nature of targeted drug resistance panels: Targeted drug resistance panels for TB are inherently dynamic, evolving in response to the ongoing discovery of novel resistance-associated mutations and the introduction of newer anti-TB drugs into clinical use. This rapidly changing landscape demands that diagnostic platforms remain adaptable and regularly updated to retain clinical relevance and diagnostic accuracy. Outdated panels risk missing emerging resistance mechanisms, which can lead to undetected resistance, inappropriate therapy, and suboptimal patient outcomes.

Crucially, it is not only the physical design of panels that must be revised, but also the bioinformatics algorithms used to detect and interpret drug resistance mutations. These computational tools must be continually updated to reflect new evidence, improve predictive performance, and accommodate changes in reference databases and mutation catalogs. Without such updates, even high-quality sequence data may yield incomplete or inaccurate resistance profiles.

To ensure continued effectiveness, real-time genomic surveillance data spanning diverse geographies and *Mycobacterium tuberculosis* lineages must be systematically integrated into both panel design and algorithmic refinement. This requires robust data-sharing pipelines, feedback loops between surveillance networks and diagnostic developers, and flexible regulatory frameworks that support iterative updates to both hardware and software components of diagnostic systems. Failure to implement such adaptive systems risks diagnostic stagnation and weakens the utility of genomics in guiding effective TB treatment and control.

Sample metadata limitations: High-quality, standardized metadata such as patient demographics, HIV status, TB treatment history, microbiological test results, drug susceptibility profiles, and geospatial and temporal information is essential to fully contextualize genomic data and extract meaningful public health insights. Such metadata is equally vital for robust bioinformatics analysis and for training reliable ML models that aim to predict drug resistance, clinical outcomes, or transmission dynamics. However, in many TB-endemic settings, metadata collection is often inconsistent, incomplete, or non-standardized, limiting the ability to link genomic data to epidemiological patterns, host factors, or treatment responses.

To maximize the utility of WGS in TB surveillance and control, it is critical to strengthen systems for metadata capture, validation, harmonization, and secure linkage to genomic datasets. This includes developing standardized metadata schemas, promoting integration with electronic medical records and laboratory information systems, and ensuring interoperability across institutions and platforms. Improving metadata quality not only enhances downstream analysis but also supports equitable participation in global TB data sharing and research efforts, ultimately leading to more targeted and effective public health interventions.

## 6. Toward Equitable Genomic Capacity

To fully realize the promise of genomics in TB control, implementation must be grounded in principles of equity, ensuring that all countries, regardless of income level, have the capacity to generate, analyze, and act upon their data. Achieving this vision requires deliberate investment in three foundational pillars, accompanied by measurable indicators to track progress.

First, decentralized and sustainable capacity building is essential. Strengthening regional and sub-national sequencing hubs, coupled with ongoing workforce development in genomics, bioinformatics, and data interpretation, can reduce reliance on external partners and enable faster, context-specific decision-making. Measurable indicators include the number of functional sequencing hubs established, the proportion of TB samples sequenced within-country, the number of personnel trained and retained in analysis roles, and average turnaround times for WGS-based results.

Second, equitable access to open-source tools, reference databases, and computational infrastructure must be prioritized. Platforms such as TB-Profiler, Mykrobe, and ReSeqTB have already democratized key components of TB WGS analysis. However, these tools must be continually adapted for use in diverse health system contexts. Indicators for monitoring include the proportion of laboratories actively using open-source genomic tools, frequency of updates and database access, bandwidth availability for data sharing, and documented instances of tool outputs incorporated into clinical or surveillance decisions.

Third, robust policy integration is critical for long-term impact. Ministries of Health and national TB programs must embed WGS into diagnostic algorithms, surveillance strategies, outbreak investigations, and public health frameworks. Progress can be measured through the inclusion of WGS in national TB strategic plans, the allocation of dedicated domestic budgets for sequencing, the establishment of data governance policies, and the number of national guidelines formally citing WGS as part of standard practice.

Without these monitoring mechanisms, WGS data risk remaining siloed within these institutions rather than informing timely, life-saving interventions. Embedding clear, measurable indicators of equity ensures that progress in TB control can be evaluated transparently and adjusted to maximize impact in high-burden LMIC settings.

## 7. Conclusions

The global fight against DR-TB stands at a pivotal crossroads. Genomic technologies, once confined to academic research, have now emerged as indispensable tools for public health enabling rapid diagnosis, real-time surveillance, and tailored treatment strategies. To effectively combat TB, particularly its drug-resistant forms, global health stakeholders must move beyond pilots and rhetoric to embed genomics into the fabric of routine clinical care and national surveillance systems.

This transformation demands more than technological readiness; it requires coordinated investment, sustained political will, and global solidarity grounded in equity. LMICs must be empowered to generate, interpret, and act on genomic data, ensuring that no region is left behind in this new era of precision public health. Integrating genomics into TB control is not merely a scientific upgrade; it is a moral imperative that strengthens resilience, saves lives, and supports more responsive, data-driven health systems.

Looking ahead, three priority research questions remain central to advancing the field: (i) how best to integrate machine learning approaches with WGS pipelines at scale to maximize diagnostic and predictive value; (ii) how to ensure the operational scalability, cost-effectiveness, and sustainability of genomic surveillance systems in LMIC contexts; and (iii) how to rigorously evaluate the impact of genomics-informed interventions on patient-level outcomes, including timeliness of diagnosis, appropriateness of therapy, and treatment success. Addressing these priorities will determine whether genomics truly fulfills its promise as a cornerstone of global TB control. 

## Figures and Tables

**Table 1:** Selected tools for TB genomic analysis, with functions, accessibility features, and limitations. Columns summarize each tool’s primary function, offline capability (ability to run without continuous internet connectivity), open-source availability, and maintenance status. These characteristics are emphasized as they are critical for sustainable implementation and routine use in low-resource, high-burden settings.

Tool (Reference)	Function	Offline Capable	Open-Source	Actively Maintained	Limitations/Scope
TBProfiler [21]	Resistance prediction, lineage	Yes	Yes	Yes	Requires regular database updates to remain accurate; limited to curated mutations.
Mykrobe [22]	Resistance + lineage	Yes	Yes	Yes	May underperform with rare/novel mutations; needs local validation in LMICs.
PhyResSE [23]	Resistance + lineage	No	Yes	Yes	Requires internet access; may not be suitable for offline/low-connectivity sites.
MTBseq [24]	Resistance + phylogenetics	Yes	Yes	Yes	Command-line expertise required; analysis can be computationally intensive.
SNP-IT [25]	Species identification	Yes	Yes	No	Not actively updated; limited scope (species ID, not resistance).
QuantTB [26]	TB mixed infections	Yes	Yes	No	Limited validation; no recent updates; may miss low-frequency minor strains.
ReSeqTB [27]	Reference database	Partial	Yes	Yes	Relies on global curation; coverage may lag behind newly emerging mutations.
IQ-TREE [28]	Phylogenetics	Yes	Yes	Yes	Requires high computational resources for large datasets; steep learning curve.
Bracken [29] and Kraken2 [30]	Contamination screening	Yes	Yes	Yes	Focuses on contamination detection; not TB-specific; large RAM needed for the reference database.

**Table 2:** Selected top-performing ML models for TB drug-resistance prediction. Performance values are reported as stated in the source publications (internal or external validation as specified by the authors). Because studies differ in datasets, endpoints, and validation design, metrics are not directly comparable across rows but illustrate the range and maturity of approaches. Model references in brackets map to the manuscript reference list.

Model and Reference	Input Features	Drugs Predicted	Performance	Key Predictors
Neural Network [48]	Demographic, clinical data, district-level FLQ resistance prevalence	Fluoroquinolones	OC-AUC-ROC = 0.87	Patient characteristics, district-level resistance prevalence
XGBoost [49]	Binary mutation features from VCF (original dataset)	Ethambutol, Isoniazid, Rifampicin	F1: EMB = 0.93, INH = 0.94, RIF = 0.92	Significant mutations from XGBC
Gradient Boosting Classifier (GBC) [46]	SNPs across 18 binary genotype matrices	Rifampicin, Isoniazid, Pyrazinamide, Ethambutol	Acc: RIF = 97.3%, INH = 96.1%, PZA = 94.2%, EMB = 92.8%	*rpoB*_Ser450, *katG*_Ser315
XGBoost [46]	WGS data (known and novel mutations)	13 anti-TB drugs	Sens: 90–95% (1st-line), 77–89% (2nd-line); Spec >95%	Known and novel resistance mutations (SHAP analysis)
Gradient Boosted Trees [50]	WGS SNPs; co-occurrence markers	14 anti-TB drugs (incl. MDR)	AUC >96% (1st-line, MDR); AUC <85% (3rd-line)	Resistance SNPs, co-occurrence markers
1D CNN [48]	Pan-genome variants (seq and non-seq features)	8 drugs incl. EMB, RIF, INH, PZA, OFX	F1: EMB = 93.8%, RIF = 96.2%, INH = 97.2%, PZA = 94.8%, OFX = 98.2%	CARD variants, 78.8% overlap with WHO catalog
Wide and Deep Neural Network [50]	Rare + known resistance variants	10 anti-TB drugs	AUC: 0.979 (1st-line), 0.936 (2nd-line)	Rare + frequent resistance-associated SNPs
ML ensemble (unspecified) [51]	WGS data from UK MTB isolates	8 drugs + MDR	Sens: up to 97% (INH, RIF, EMB), 96% (MDR), 95–96% (MOX, OFX)	SNPs outperforming rules-based predictions

## Data Availability

No new data were created.

## Abbreviations

The following abbreviations are used in this manuscript:

**Abbrev.**	**Full Term**	**Definition/Notes**
ANN	Artificial Neural Network	Machine-learning model inspired by biological neurons; learns complex nonlinear mappings.
AUC	Area Under the Curve	Usually area under the ROC curve; scalar measure of discrimination (higher is better).
CARD	Comprehensive Antibiotic Resistance Database	Curated resource of known antimicrobial resistance genes/mutations.
CNN	Convolutional Neural Network	Deep-learning architecture well-suited to spatial/structured data (e.g., images; can be adapted to genomics).
EMB	Ethambutol	First-line anti-tuberculosis drug.
FLQ	Fluoroquinolones	Class of broad-spectrum antibiotics; key second-line drugs in TB treatment.
F1	F1-score	Harmonic mean of precision and recall; balances false positives/negatives in classification.
GBC	Gradient Boosting Classifier	Ensemble of shallow decision trees trained sequentially to correct prior errors.
INH	Isoniazid	First-line anti-tuberculosis drug.
RIF	Rifampicin	First-line anti-tuberculosis drug.
PZA	Pyrazinamide	First-line anti-tuberculosis drug.
MDR	Multidrug-Resistant	Resistance to at least isoniazid and rifampicin.
ML	Machine Learning	Methods that learn patterns from data to make predictions/decisions.
MOX	Moxifloxacin	Fluoroquinolone antibiotic.
MTB	Mycobacterium tuberculosis	Bacterial species that causes most human TB.
OC-AUC-ROC	Optimism-Corrected AUC-ROC	AUC adjusted for overfitting (e.g., via bootstrap/cross-validation).
OFX	Ofloxacin	Fluoroquinolone antibiotic.
rpoB_Ser450	rpoB Ser450 mutation	Canonical rifampicin-resistance mutation (serine at codon 450) in rpoB.
katG_Ser315	katG Ser315 mutation	Canonical isoniazid-resistance mutation (serine at codon 315) in katG.
Sens	Sensitivity	True-positive rate; ability to correctly identify resistant cases.
Spec	Specificity	True-negative rate; ability to correctly identify susceptible cases.
SHAP	SHapley Additive exPlanations	Game-theoretic method to explain feature contributions in ML models.
SNP	Single Nucleotide Polymorphism	Single-base DNA variation among individuals.
VCF	Variant Call Format	Standard text format for storing sequence variants.
WGS	Whole Genome Sequencing	Determination of the complete DNA sequence of an organism’s genome.
WHO	World Health Organization	United Nations specialized agency for international public health.
